# The Type III Effectome of the Symbiotic *Bradyrhizobium* *vignae* Strain ORS3257

**DOI:** 10.3390/biom11111592

**Published:** 2021-10-28

**Authors:** Nicolas Busset, Djamel Gully, Albin Teulet, Joël Fardoux, Alicia Camuel, David Cornu, Dany Severac, Eric Giraud, Peter Mergaert

**Affiliations:** 1 Université Paris-Saclay, CEA, CNRS, Institute for Integrative Biology of the Cell (I2BC), F-91198 Gif-sur-Yvette, France; nicolas.busset@ird.fr (N.B.); david.cornu@i2bc.paris-saclay.fr (D.C.); 2 Laboratoire des Symbioses Tropicales et Méditerranéennes (LSTM), UMR IRD/SupAgro/INRAE/Université de Montpellier/CIRAD-Campus de Baillarguet, F-34398 Montpellier, France; djamel.gully@ird.fr (D.G.); albin.teulet@slcu.cam.ac.uk (A.T.); Joel.Fardoux@Ird.Fr (J.F.); camuel.alicia@gmail.com (A.C.); 3 Institut de Génomique Fonctionnelle, Université Montpellier, CNRS, INSERM, F-34094 Montpellier, France; dany.severac@mgx.cnrs.fr; 4 Montpellier GenomiX, France Génomique, F-34094 Montpellier, France

**Keywords:** *Bradyrhizobium*, type III secretion system, effector, nodulation, legume symbiosis, nod factor, proteome, transcriptome

## Abstract

Many *Bradyrhizobium* strains are able to establish a Nod factor-independent symbiosis with the leguminous plant *Aeschynomene indica* by the use of a type III secretion system (T3SS). Recently, an important advance in the understanding of the molecular factors supporting this symbiosis has been achieved by the in silico identification and functional characterization of 27 putative T3SS effectors (T3Es) of *Bradyrhizobium vignae* ORS3257. In the present study, we experimentally extend this catalog of T3Es by using a multi-omics approach. Transcriptome analysis under non-inducing and inducing conditions in the ORS3257 wild-type strain and the *ttsI* mutant revealed that the expression of 18 out of the 27 putative effectors previously identified, is under the control of TtsI, the global transcriptional regulator of T3SS and T3Es. Quantitative shotgun proteome analysis of culture supernatant in the wild type and *T3SS* mutant strains confirmed that 15 of the previously determined candidate T3Es are secreted by the T3SS. Moreover, the combined approaches identified nine additional putative T3Es and one of them was experimentally validated as a novel effector. Our study underscores the power of combined proteome and transcriptome analyses to complement in silico predictions and produce nearly complete effector catalogs. The establishment of the ORS3257 effectome will form the basis for a full appraisal of the symbiotic properties of this strain during its interaction with various host legumes via different processes.

## 1. Introduction

Bradyrhizobia are Gram-negative bacteria that are widespread and dominant in soils all around the world [1]. The best-known *Bradyrhizobium* strains are symbiotic nitrogen fixers, interacting with a large diversity of legumes, including plants of agronomic interest [2]. This symbiosis leads to the formation of new root organs, the nodules, in which the bacteria differentiate into a nitrogen-fixing form called bacteroids. The major part of the nitrogen fixed by the bacteroids is transferred to the plant, which can therefore grow in soils that are deficient in available nitrogen for the plant. In exchange, bacteria obtain a privileged ecological niche within the nodules where they can massively multiply thanks to nutrients provided by the plant, including reduced carbon resulting from photosynthesis.

This symbiotic process between the two partners is regulated by the exchange of signal molecules. One of the key steps allowing the initiation of symbiosis is the secretion by the bacteria of lipochitooligosaccharide signals called Nod factors (NFs), whose synthesis is induced by the perception of plant flavonoids [3]. The recognition of NFs by the host plant leads to the activation of a signaling cascade regulating two coordinated processes, nodule organogenesis and its infection by rhizobia, thus allowing the formation of a functional nodule [4]. This Nod factor-dependent symbiotic process was considered universal for all rhizobium-legume interactions until the identification of a specific clade of photosynthetic *Bradyrhizobium* strains that lack the genes crucial for the NF synthesis but that are, nevertheless, able to establish a functional symbiosis with tropical legumes of the genus *Aeschynomene* [5]. This iconoclastic finding demonstrated the existence of non-conventional symbiotic processes.

More recently, it was shown that other *Bradyrhizobium* species can nodulate some legumes, even when their NF synthesis is abolished by mutagenesis or when the perception of NF in the host plant is inactivated. The *Bradyrhizobium elkanii* strain USDA61 can nodulate *Glycine max* cv. Enrei (soybean) as well as *Aeschynomene indica* in the absence of NFs but in a Type III Secretion System (T3SS)-dependent manner [6,7,8]. Similarly, the *Bradyrhizobium vignae* strain ORS3257 requires its T3SS to induce nodules on the NF-independent legume *A. indica* [7,9]. This T3SS-dependent nodulation of *A. indica* is distinct from the NF-independent process employed by the earlier described photosynthetic brady-rhizobia that produce no NFs but also have no T3SS and thus depend on a different signaling mechanism, which still remains unknown [5,10].

The T3SS was first studied in animal pathogens like bacteria of the genus *Shigella*, *Yersinia*, *Salmonella*, and *Pseudomonas*, in which it is a key pathogenesis factor [11]. The T3SS functions as a nano-syringe that enables the bacteria to inject proteins, called effectors, from its cytosol directly into neighboring host cells. The delivered effectors then interfere with cellular processes such as the cytoskeleton organization and the immune system, thereby promoting pathogenesis [12]. Similarly, phytopathogenic bacteria like *Pseudomonas*, *Xanthomonas*, *Erwinia*, and *Burkholderia* species use T3SS effectors (T3Es) to inhibit the so-called PAMP (pathogen-associated molecular pattern)-triggered immunity or PTI in plants. These T3Es are the subject of a cat-and-mouse game between pathogen and host plant as they can also be recognized by plant immune receptors (resistance (R) proteins), leading to the activation of another layer of defense, called effector-triggered immunity or ETI [13]. Interestingly, many rhizobia also possess a T3SS and use T3Es to interfere with plant immunity and increase their symbiotic efficiency and their host spectrum. However, similarly as observed for phytopathogens, some T3Es can be recognized in other hosts by R proteins triggering an ETI that blocks the symbiosis [14,15].

**Figure 1:**
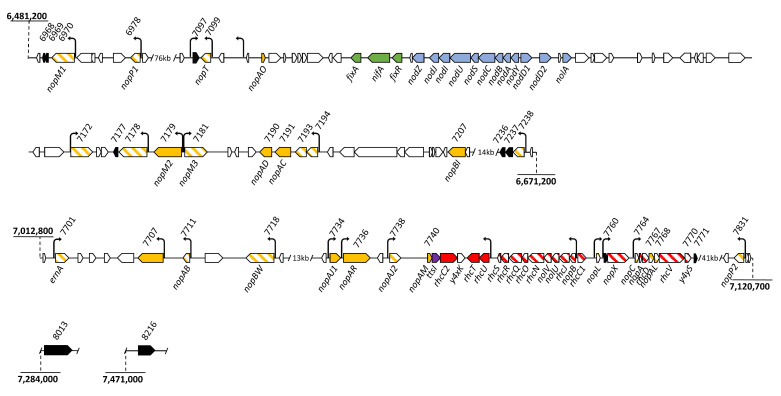
Genetic organization of *Bradyrhizobium* ORS3257 symbiotic island encoding the Type III secretion system and nodulation genes. Green, *nif*, and *fix* genes involved in nitrogen fixation; blue, *nod* genes; violet, *ttsI*; yellow, putative effector genes previously identified by in silico prediction; red, genes encoding components of the T3SS apparatus; black, new putative effectors; black arrows, *tts*-boxes; hatched boxes represent genes regulated by TtsI. Adapted from [9].

The NF-independent but T3SS-dependent nodulation in some soybean cultivars and in *Aeschynomene* by bradyrhizobia, suggests an additional role of the T3SS in these strains, enabling a symbiotic process in which the NF-signaling is replaced by T3SS-signaling [6,7,8,9]. A previous study, based on in silico predictions, coupling the search for *tts*-box motifs to which the TtsI regulator of rhizobial T3SS binds and the identification of homologs with known T3Es, revealed 27 putative *T3E* genes in the *B. vignae* strain ORS3257 genome [9]. They were all located within two distinct regions of the symbiotic island, one containing the nodulation (*nod*) genes and the other one containing the genes encoding the T3SS machinery (Figure 1). A broad survey of effectors in nodulating *Bradyrhizobium* strains concluded that nearly all T3Es in these bacteria are located within the symbiotic islands, suggesting a strong functional link of the T3SS with the nodulation process in these bacteria [10]. Twenty-three of the predicted T3E-encoding genes in strain ORS3257 were mutated to characterize their role during symbiosis with *A. indica* [9]. This analysis highlighted that several T3Es play roles at different stages of the symbiotic process, such as the formation of nodules, their infection, or the dampening of plant immunity to permit a chronic infection. In particular, a new effector named ErnA was identified that triggers the nodulation process.

Since the in silico analysis revealed, on the one hand, T3E candidates with homology to known effectors but without upstream *tts*-box in their corresponding gene, and on the other hand, *T3E* genes with *tts*-box but without homology to known effectors, it is not excluded that additional T3Es are encoded in the genome of strain ORS3257 that have no *tts*-box and no homology. Furthermore, this in silico approach is only predictive and can lead to an overestimation of the number of T3Es. Therefore, revealing the complete set of effectors in strain ORS3257 with experimental methods is of cardinal importance to study the full potential of the effector arsenal, the effectome, to interfere with host signaling during nodulation. The aim of the present study was to validate and expand the T3E catalog identified in silico in strain ORS3257 by combining a transcriptome approach to identify the set of genes under the control of the TtsI regulator with a proteome analysis to determine the proteins secreted in a T3SS-dependent manner by ORS3257. This multi-omics approach experimentally validated a majority of previously predicted T3Es and identified a new one containing a ubiquitin-like protease (ULP)-like domain.

## 2. Materials and Methods

### 2.1. Bacterial Strains and Growth Conditions

The *B. vignae* strain ORS3257 and its derivatives (Table S1, see Supplementary Materials) were grown in yeast mannitol (YM) medium at 28 °C or 34 °C. *Escherichia coli* strains were grown at 37 °C in Lysogeny broth (LB) medium. When required, the media were supplemented with the appropriate antibiotics at the following concentrations: 50 µg/mL kanamycin (Km), 20 µg/mL nalidixic acid (Nal), 20 µg/mL cefotaxime (Cefo), and 20 µg/mL spectinomycin (Sp).

### 2.2. Plasmid Construction and Mutagenesis

All the constructs made in this study are listed in Table S1 (see Supplementary Materials), which also includes the primers and the cloning strategies. For the construction of the deletion mutants by double crossing-over, 750 to 1000 bp PCR fragments corresponding to the upstream and downstream flanking regions of the gene or locus to delete were merged by overlap extension PCR and cloned into pNPTS129, which carries the *sacB* gene. The resulting plasmids were then transferred by mating into the ORS3257 strain, and deletion mutants were obtained by counter selection of the *sacB* gene on plates containing 10% sucrose, as described previously [16]. For the construction of insertional mutants by single crossing-over, 250 to 400-bp internal fragments of the gene to mutate were amplified by PCR and were cloned into the non-replicative plasmid pVO155-npt2-GFP-npt2-Cefo [7]. The plasmids were transferred by mating into the ORS3257 strain, resulting in their integration in the genome by single crossing-over and interruption of the targeted gene.

For the construction of the *ttsI* overexpressor strains, the complete open reading frame was cloned into the non-replicative plasmid pVO155-npt2-GFP-npt2-Cefo under the control of the *npt2* promoter [7]. The plasmid was transferred by mating into the ORS3257 wild-type (WT) and ORS3257∆*T3SS* strains. The integration of the plasmid in the genome by a single crossing-over resulted in a merodiploid strain carrying two copies of the *ttsI* gene, one under the control of its native promoter and a second copy under the control of the constitutive and strong *npt2* promoter.

The ORS3257 WT and ORS3257∆*T3SS* strains expressing His_6_-tagged proteins were constructed by cloning a fragment encompassing the complete *BRAD3257_v2_7792*, *BRAD3257_v2_8013*, or *BRAD3257_v2_8216* gene and their upstream promoter regions using reverse primers containing a His_6_-tag sequence. These inserts were then cloned into the *Xba*I or *Xho*I site of pVO155-npt2-GFP-npt2-Cefo and introduced into the ORS3257 WT and ORS3257∆*T3SS* strains by single crossing-over. The resulting merodiploid strains carried two copies of the putative effector genes, both under the control of the native promoter, one encoding the WT protein and the other the His_6_-tagged protein. All the constructed strains were verified by PCR to confirm their identity.

### 2.3. Transcriptome Analysis

*B. vignae* ORS3257 WT strain and its Ω*ttsI* mutant [9] were cultivated in BNM-B minimal medium at 34 °C [17]. When the OD_600_ reached ~0.4, 5 μM of genistein dissolved in DMSO or DMSO alone was added and the cultures were harvested after 24 h. Total RNA was extracted using the Ribopure Bacteria kit (Ambion), treated with DNase I (Qiagen), and submitted to bacterial rRNA depletion using the RiboZero kit for Gram-negative bacteria (Illumina Inc., Paris, France). Each condition was performed in independent biological duplicates. The eight corresponding RNA-seq libraries were generated and sequenced using HiSeq2000 technology (single reads 50bp; Illumina Inc., USA). For each sample, 32 to 41 million reads were obtained, out of which more than 92% were assigned to mRNA. The sequencing data of this project was submitted to the Sequence Read Archive (SRA) and obtained accession n° SRR14479858 to SRR14479861. The transcriptomic data were analyzed as previously described [18], using the ORS3257 genome as a reference [19]. The differential expression level of each gene between conditions was determined using the DESeq2 statistical analysis [20]. Quality data of the RNA-seq are shown in Figure S1 (see Supplementary Materials).

### 2.4. Proteome Analysis

For isolation of extracellular proteins, bacteria were grown in 600 mL of BNM-B minimal medium [17] inoculated at 1:200 with precultures of ORS3257 WT or ORS3257∆*T3SS* strains and their derivatives overexpressing TtsI (See Table S1 in Supplemental Materials for the constructions). Bacteria were cultivated in the presence of 5 µM genistein at 28 °C and 200 rpm for 48 h. Supernatants were recovered from the bacterial cultures by two rounds of centrifugation (4000× *g* at 4 °C for 1 h and 8000× *g* at 4 °C for 30 min). Supernatants were lyophilized, and extracellular proteins were extracted as described previously [21].

For mass spectrometry analysis, proteins from each condition were successively resuspended in Rapigest (Waters), reduced/alkylated with DTT and iodoacetamide and subjected to trypsin digestion at 37 °C (overnight). After hydrolysis of Rapigest (protocol from Waters), tryptic peptide solutions were vacuum dried and resuspended in solvent A (0.1% (*v/v*) formic acid). Tryptic peptides from different conditions were analyzed by nanoLC-MS/MS with the Triple-TOF 4600 mass spectrometer (ABSciex) coupled to the nanoRSLC ultra-performance liquid chromatography (UPLC) system (Thermo Scientific) equipped with a trap column (Acclaim PepMap100C18, 75 μmi.d. × 2 cm, 3 μm) and an analytical column (Acclaim PepMapRSLCC18, 75 μmi.d. × 50 cm, 2 μm, 100 Å). Peptides were loaded at 5 µL/min with 0.05% TFA in 5% acetonitrile, and peptide separation was performed at a flow rate of 300 nL/min with a 5 to 35% solvent B (0.1% formic acid in 100% acetonitrile) gradient in 40 min. NanoLC-MS/MS experiments were conducted in a data-dependent acquisition method by selecting the 20 most intense precursors for CID fragmentation with Q1 quadrupole set at low resolution for better sensitivity.

For protein identification, raw data were processed with the MS Data Converter software (AB Sciex) for generating.mgf data files, and protein identification was performed using the MASCOT search engine (Matrix science, London, UK) against the ORS3257 database with carbamidomethylation of cysteine residues set as a fixed modification and oxidation of methionine residues as a variable modification. Peptide and fragment tolerance were respectively set at 20 ppm and 0.05 Da. Proteins were validated when identified with at least two unique peptides and only peptides with Mascot ion scores above identity threshold at less than 1% FDR (false discovery rate) were considered [22]. This analysis was performed in triplicate.

Predictions of the cellular localization of proteins were carried out using the tools available on the MicroScope platform (https://mage.genoscope.cns.fr/microscope/, accessed on 27 October 2021). The ULP domain was inspected using NCBI Conserved Domain Search (https://www.ncbi.nlm.nih.gov/Structure/cdd/wrpsb.cgi, accessed on 27 October 2021). Repeat domains were identied using Tandem Repeats Finder (https://tandem.bu.edu/trf/trf.html, accessed on 27 October 2021). NLSs were predicted using NLS Mapper (http://nls-mapper.iab.keio.ac.jp/cgi-bin/NLS_Mapper_form.cgi, accessed on 27 October 2021). SUMO-protease domains of BRAD3257_v2_7792, Bel2–5 (*B. elkanii* strain USDA61) and NopD (*Bradyrhizobium* sp. XS1150) were aligned using the MUSCLE algorithm.

### 2.5. Western Blot Analysis

Six hundred milliliters of BNM-B minimal medium were inoculated at 1:200 with precultures of ORS3257 or ORS3257∆*T3SS* strains expressing a His_6_-tagged version of the putative effectors (Table S1, see Supplementary Materials). Bacteria were cultivated in the presence of 5 µM genistein at 28 °C for 48 h at 200 rpm. Bacterial cells and exopolysaccharides were separated from the supernatant by two centrifugation steps (first step: 1 h, 4000× *g*, 4 °C; second step: 30 min, 8000× *g*, 4 °C). Proteins in the supernatant were precipitated using trichloroacetic acid as previously described [23] and resuspended in 75 µL of NuPAGE™ LDS Sample Buffer (Thermofischer, Illkirch, France) for SDS-PAGE analysis. Bacterial cells were resuspended in 5 mL of solubilization buffer (50 mM Tris-HCl pH 8, 20 mM imidazole and 300 mM NaCl) and lysed by 5 freeze-thaw cycles in liquid nitrogen. Cell debris was removed by centrifugation (30 min, 21,000× g, 4 °C), and LDS Sample Buffer 4x was added to the supernatants for SDS-PAGE analysis.

Twenty-five µL protein solutions were separated on 12.5% SDS-PAGE gels and transferred to PVDF membranes. The membranes were then blocked using PBSTM consisting of 5% non-fat milk in PBST buffer (1× PBS with 0.1% Tween 20). Anti-His_6_ Mouse Antibodies (1:1000; #SAB1305538; Merck) were added to the PBSTM and the membranes were incubated 2 h at room temperature. Then, the membranes were incubated for 2 h with peroxidase-conjugated anti-mouse antibodies (1:500; #A9044; Merck). Revelation of Western blots was performed by chemiluminescence using Pierce™ ECL Plus Western Blotting Substrate (#32132; ThermoFischer) according to the manufacturer’s protocols. 

### 2.6. Symbiotic Tests

Culture and rhizobial inoculation of *A. indica* plants were performed as previously described [7]. For nodulation and the nitrogen-fixation assay, 12 plants per condition at 21 days post-inoculation (dpi) were analyzed for quantification of the number of nodules formed and for determination of the nitrogenase activity by the acetylene reduction assay, as previously described [9]. The experiments were carried out in duplicate. Microscopy analysis was performed on fresh nodules, sectioned with a Leica VT1200S vibratome (Leica Microsystems GmbH) into 30 µm tissue slices. The nodule sections were incubated 15 min in live/dead staining solution and were analyzed as previously described [7].

### 2.7. Outer Membrane Vesicles Observation by Transmission Electron Microscopy

Precultures grown in YM medium of the *B. vignae* strain ORS3257 and its derivatives were inoculated at 1:200 in 20 mL half-strength YM and cultures were grown at 28 °C for 3 days. Ten µL of the cultures were deposited on a microscopy grid. After 5 min, the excess liquid was removed with a filter paper and then 10 μL of 0.5% uranyl acetate was deposited for staining for one minute. After removing the liquid on the grids with filter paper, they were observed using a STEM JEOL 1400 microscope.

## 3. Results

### 3.1. Transcriptome Identification of the Genes under the Control of TtsI and the Flavonoid Genistein in Bradyrhizobium vignae ORS3257

In rhizobia, the expression of the T3SS machinery and T3Es is under the control of the TtsI transcriptional regulator, which is in turn induced by the NodD transcriptional activator and plant-derived flavonoids [24]. TtsI is activated in strain ORS3257 by the flavonoid genistein [9]. In order to identify the genes under the control of TtsI and genistein, a transcriptome analysis was carried out on in vitro cultures of ORS3257 WT and the ORS3257Ω*ttsI* mutant strain in the presence or absence of the inducer genistein. 

The comparison of the gene expression between the WT and ORS3257Ω*ttsI* in the presence of genistein revealed the overexpression (fold change ≥ 2, *p*-value < 0.01) of 61 coding sequences out of 8969 in the WT strain compared to the mutant (Table S2, see Supplemental Materials). Out of these 61, 40 genes were significantly induced (fold change ≥ 2, *p*-value < 0.01) by genistein, and their genistein-induction was dependent on TtsI.

#### 3.1.1. T3SS Machinery and Predicted T3SS Effectors Genes Regulated by TtsI

**Figure 2:**
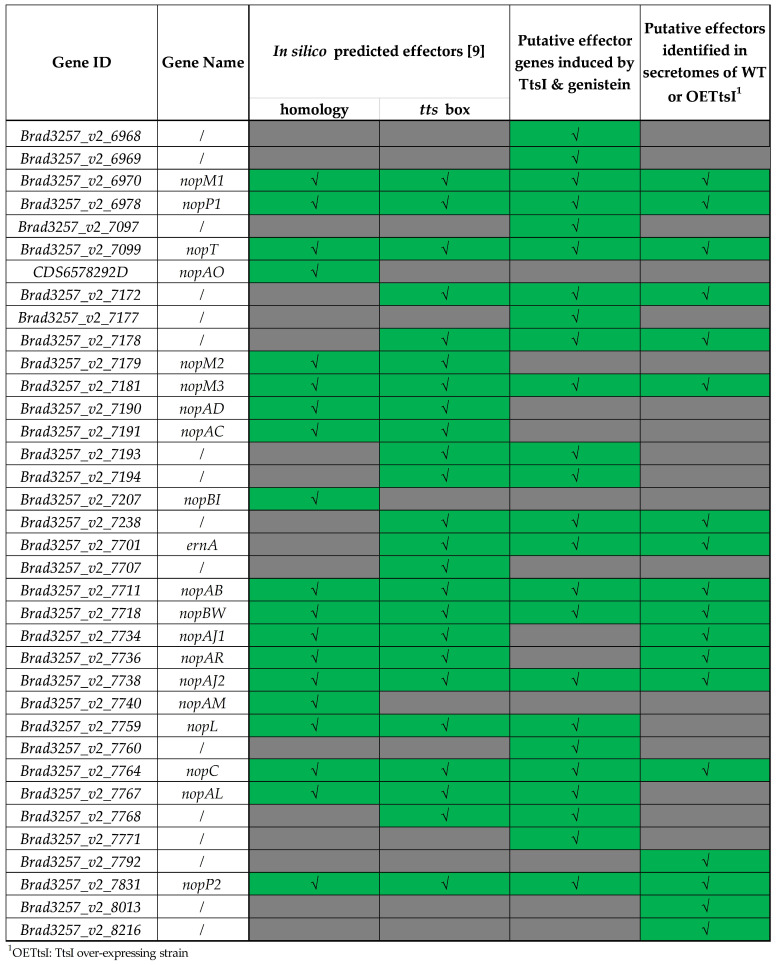
Overview of putative effectors in *Bradyrhizobium vignae* strain ORS3257.

In the genome of the ORS3257 strain, 18 genes involved in the synthesis of the T3SS machinery are known and grouped within the symbiotic island [9]. Among them, 14 genes (*nopBrhcJnolUVrhcNOQRS, nopX, nopArhcDVy4yS*), located in three transcriptional units carrying a *tts*-box in the upstream region, were upregulated in the WT condition with respect to the *ttsI* mutant, confirming that their expression is under the control of TtsI and in agreement with the presence of a *tts*-box (Figure 1 and Figure 2). In addition, we noticed that the *rhcC1* gene, which is adjacent to the *nopB* operon but transcribed in the opposite orientation, is also upregulated by genistein in a TtsI-dependent fashion (Table S2), despite the fact that the *tts*-box is oriented towards the *nopB* operon (Figure 1). This expression pattern suggests that this *tts*-box controls expression in both orientations. Twelve of these genes code for the basal body of the T3SS machinery and the three others for the needle (*nopA*, *nopB*) and the translocator (*nopX*). In contrast, the expression of the genes *rhcC2*, *rhcU*, and *rhcT*, encoding additional T3SS components, seemed not to be dependent on TtsI in the tested conditions of the transcriptome analysis (Table S3), despite the presence of a *tts*-box upstream of the *rhcUT* operon. These results confirm that the transcriptome analysis allows the identification of genes under the control of TtsI, but also suggest that the presence of a *tts*-box is not an absolute indicator of TtsI-dependent expression or that a *tts*-box can direct expression in the opposite orientation. Additional experiments using reporter gene fusions or RT-qPCR with more genistein-induction times could be used to test the latter hypotheses in more detail.

Among the 27 previously identified putative effector genes, 18 are overexpressed in the WT-strain compared to the *ttsI* mutant (Figure 1 and Figure 2), suggesting that their transcription is under the control of TtsI. Among them, seven encode the common rhizobial effectors NopM1, NopP1, NopT, NopM3, NopL, NopC, and NopP2, four encode the *Bradyrhizobium*-specific effectors NopAB*,* NopBW*,* NopAJ2, and NopAL, and seven correspond to putative novel effectors predicted in our previous study (Figure 1 and Figure 2). In addition, two putative effectors, encoded by *Brad3257_v2_7707* and *Brad3257_v2_7734* (*nopAJ1*), are potentially also under the control of TtsI, but their fold change or associated *p*-value fell below the selected cutoff values (Table S3). All the above putative effector genes have an upstream *tts*-box, consistent with their TtsI-dependent expression. However, the expression of four additional putative effector genes, *nopM2* (*Brad3257_v2_7179*), *nopAD* (*Brad3257_v2_*7190), *nopAC* (*Brad3257_v2_7191*), and *nopAR* (*Brad3257_v2_7736*), each possessing a *tts-box* motif in their upstream region, was not dependent on TtsI in the tested conditions (Table S3). The three remaining predicted effector genes, *nopAO* (*CDS6578292D*), *nopBI* (*Brad3257_v2_7207*), and *nopAM* (*Brad3257_v2_7740*), do not have an upstream *tts-box* motif and were found not to be regulated by TtsI (Figure 1; Tables S2 and S3, see Supplementary Materials).

#### 3.1.2. Identification of New Putative Effectors by Transcriptome Analysis

Among the open reading frames (ORFs) overexpressed in the WT condition compared to the *ttsI* mutant, 12 are scattered all over the ORS3257 genome and are located outside of the symbiotic island (Table S2, see Supplementary Materials). Ten ORFs are conserved bacterial genes encoding transcription factors, metabolic proteins, or components of the flagellar machinery and are therefore unlikely effectors. The last two genes, *BRAD3257_v2_1638* and *BRAD3257_v2_4946* do not have homologies with known proteins, and *BRAD3257_v2_1638* is even specific for strain ORS2357. However, because of the prevalent location of effectors in the symbiotic island, these last two genes were not further considered.

All the remaining TtsI-dependent genes are located within the symbiotic island. Unexpectedly, among them are the five nodulation genes, *nodY*, *nodA*, *nodB*, *nodC*, and *nodS*, coding for the synthesis of NFs (Table S2, see Supplementary Materials). The regulation of nodulation genes in bradyrhizobia is complex, involving a network of several regulators. In *B. diazoefficiens* USDA110, for which the mechanisms of nodulation gene regulation are the best described, this includes the two NodD proteins (NodD1 and NodD2), which act antagonistically, the two-component regulatory system NodV/NodW, which activates the *nodD1* and *nodYABCS* genes and the NolA regulator which activates *nodD2* [25]. But to our knowledge, the regulation of nodulation genes by the TtsI regulator was not reported before. Our transcriptome data showed that the *nodYABCS* genes are upregulated by genistein in the WT, in agreement with the presence of a well-conserved *nod*-box upstream of *nodY* and NodD regulation [25]. In contrast, no genistein induction of these genes was observed in the *ttsI* mutant background. Thus, TtsI appears to be an additional crucial regulator of the nodulation genes in strain ORS2357, despite the absence of a *tts*-box upstream of *nodY* or the two *nodD* genes. However, the details of this regulation as well as the interplay with the other regulators of the strain require further investigation.

Seven other coding sequences (CDS) are present in the symbiotic island (Table S2, see Supplementary Materials). Among them, two genes (*Brad3257_v2_6968* and *Brad3257_v2_6969*) with a small size, 195 and 291 bp, are downstream of *nopM1*, which is preceded by a *tts*-box (Figure 1). The *Brad3257_v2_6968* gene encodes an uncharacterized protein that is conserved among many bradyrhizobia but is not found outside of this clade. The *Brad3257_v2_6969* CDS corresponds to a protein of unknown function specific to ORS3257. The CDS, *Brad3257_v2_7236* and *Brad3257_v2_7237*, correspond to fragments of the neighboring putative effector gene *Brad3257_v2_7238*, which displays homology with trehalose-6-phosphate synthase effectors of plant pathogens [26] and which was predicted by the presence of a *tts*-box and validated by our transcriptome data. 

Finally, the transcriptome analysis pointed out five additional candidate T3SS effector genes (Table S2, see Supplementary Materials; Figure 1 and Figure 2). These genes include *Brad3257_v2_7097* near *nopT* but orientated in the opposite direction and having a *tts*-box in its promoter region, *Brad3257_v2_7177* downstream of the predicted effector gene *Brad3257_v2_7178* and possibly under the control of the same promoter, *Brad3257_v2_7760* located just upstream of the *nopX* gene and preceded by a *tts*-box, and *Brad3257_v2_7771* encoding a small protein of 52 amino acids. The latter gene is probably part of a large operon, starting with *Brad3257_v2_7764* (*nopC*) and encoding both T3SS machinery components and putative effectors.

In summary, the gene expression analysis demonstrated the TtsI-dependent expression for 18 (or 20 if *Brad3257_v2_7707* and *Brad3257_v2_7734* (*nopAJ1*) are included despite falling below the set cutoffs for significant differential expression) predicted effectors and identified up to 6 new potential effectors (*Brad3257_v2_6968*, *Brad3257_v2_6969*, *Brad3257_v2_7097*, *Brad3257_v2_7177*, *Brad3257_v2_7760*, and *Brad3257_v2_7771*) located within the symbiotic island. However, these additional putative effectors remain speculative considering that they could correspond to fragments of degenerating genes and that the largest encoded protein among them is less than 100 amino acids.

### 3.2. T3SS-Dependent Secretome in Bradyrhizobium vignae ORS3257

In order to identify the proteins secreted in a T3SS-dependent manner by the ORS3257 strain, a liquid chromatography coupled to mass spectrometry (LC-MS) shotgun proteome analysis was performed on the culture supernatant of bacteria grown in the presence of genistein to induce the expression of the T3SS machinery and *T3Es* genes. The analysis was carried out on the WT strain and a T3SS deletion mutant (∆*T3SS*), as well as the corresponding strains that over-express the TtsI regulator to increase the production and secretion of effectors in the culture medium. 

#### 3.2.1. Cytosolic and Membrane Proteins Are Present in the Supernatant

**Figure 3:**
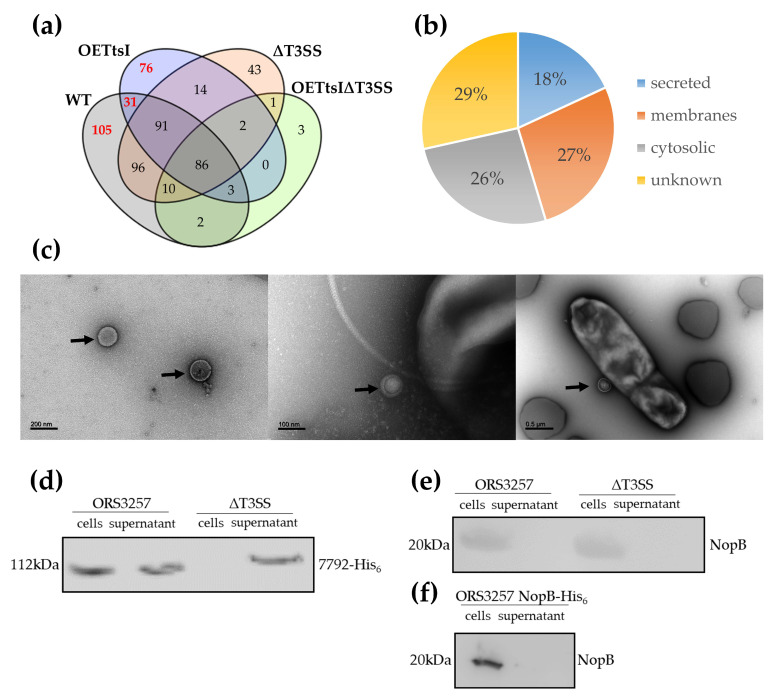
T3SS-dependent secretome in *Bradyrhizobium vignae* ORS3257. (**a**) Venn diagram of secreted proteins by ORS3257 WT and T3SS mutant strains. OETtsI is the ORS3257 strain overexpressing TtsI. ∆T3SS carries a deletion of the *rhcN* gene, resulting in a non-functional T3SS. In red, proteins not found in the supernatant of the T3SS mutants, which are candidate T3SS effectors. (**b**) Origin of proteins identified in the supernatant of ORS3257 and its T3SS mutant derivative. (**c**) Observation by negative staining using a transmission electron microscope of the ORS3257 WT (left and middle), and the ORS3257 OETtsI (right) strains in liquid culture. Arrows indicate OMVs. (**d**) Western blot analysis of the BRAD3257_v2_7792 effector in cell-associated and supernatant proteins of ORS3257 WT and ∆T3SS strains with an anti-His_6_ antibody. (**e**) Western blot analysis of the NopB T3SS-needle protein in cell-associated and supernatant proteins of ORS3257 WT and ∆T3SS strains with an anti-NopB antibody. (**f**) Western blot analysis of NopB in cell-associated and supernatant proteins of ORS3257 WT with an anti-His_6_ antibody.

The total number of proteins identified in the culture supernatants for the four conditions combined, validated with the identification of at least two unique peptides, is 563 (Figure 3a; Table S4, see Supplementary Materials). These proteins included predicted secreted proteins (101), unknown proteins without a clear, predictable localization (159), as well as many proteins predicted to be located in the membranes (152) or the cytosol (146) of the bacteria (Figure 3b). It is common to find cellular proteins in the supernatant due to incomplete removal of cells, but the centrifugation protocol used to separate the bacterial cells from the supernatant minimized this issue. An alternative source of this type of protein in the culture supernatants is the production of outer membrane vesicles (OMV) [27]. Indeed, the protocol used to remove bacterial cells from the culture supernatants does not eliminate OMVs, which are then lysed during protein precipitation of the supernatants. Observations by negative staining using a transmission electron microscope of ORS3257 and T3SS mutant cultures revealed the presence of OMVs in all cultures, indicating that at least some of the identified cellular proteins in the proteomic analyses can originate from these vesicles (Figure 3c).

Proteins commonly found in OMV of many species of bacteria such as *E. coli*, *Pseudomonas syringae*, or *Helicobacter pylori* are registered in the Vesiclepedia repository (http://microvesicles.org, accessed on 27 October 2021) [28]. The comparison of proteins present in the supernatant of the different conditions of this analysis with the proteins identified in OMV of other bacterial species showed that 182 identified membrane or cytosolic proteins could originate from OMVs. In addition, it is possible that *Bradyrhizobium*-specific proteins, which are therefore absent from this database, are also present in OMVs (Table S4, see Supplementary Materials). 

#### 3.2.2. The Expression Level of the T3SS Apparatus Modulates Protein Production and Secretion in *Bradyrhizobium vignae* ORS3257

The protein content of the supernatants of the WT and *ttsI*-overexpressing strains diverge by more than half of the proteins and only 211 are commonly present, out of a total of 516 proteins detected in the supernatants of these two strains (Figure 3a). This variability is also observed in the comparison of the supernatants of the WT strain and the Δ*T3SS* mutant, but to a lesser extent with 283 common proteins out of 484 (Table S4, see Supplementary Materials). A large portion of the variable proteins is derived from the cytosol or bacterial membranes (Table S4, see Supplementary Materials). It is known that the T3SS synthesis and activity has an important energetic cost for the bacterial cell, hence the synthesis of the T3SS machinery and the expression of effectors are under the control of inducible promoters such as TtsI for rhizobial strains [24,29]. Therefore, we propose that the variability between conditions is an indirect effect resulting from changes in bacterial metabolism due to the energy cost of expression or overexpression of the T3SS machinery. Moreover, since TtsI is a regulatory protein, it is possible that its overexpression has unexpected effects, altering the expression of cellular processes that are not directly under its control in WT cells. 

#### 3.2.3. T3SS-Dependent Secreted Proteins by *Bradyrhizobium vignae* ORS3257 and Identification of New Putative Effectors

The candidate Type III effectors are expected to be found in the WT conditions with or without overexpression of TtsI and absent from the supernatant of the T3SS mutants. This is the case for 212 proteins, but only 31 of them are common to the WT and TtsI-overexpressing strain (Figure 3a; Table S4, see Supplementary Materials). Satisfyingly, all the candidate effectors determined by prediction and transcriptome analysis and detected in the proteome analysis were found in the supernatant of WT or the TtsI-overexpressing strain and were absent from the supernatant of the T3SS mutants (Figure 2; Table S4, see Supplementary Materials). Among the 212 proteins, 51 are membrane proteins, 58 have a cytosolic origin and 25 are secretory proteins but their secretion is predicted to be in a T3SS-independent manner. These proteins can be eliminated from candidates to be T3Es, leaving 78 proteins to be further considered (Table S4, see Supplementary Materials). Among them, besides the predicted effectors, 62 proteins whose cellular localization is unknown, were detected. However, the majority of the genes encoding these proteins are located outside the symbiotic island, excluding them from consideration.

The 18 remaining proteins, present only in the supernatant of the WT strain and its TtsI-overexpressing derivative and not in the corresponding T3SS mutants, are putative effectors (Figure 2; Table S4, see Supplementary Materials). It is useful to note that some putative effectors were only detected when TtsI is overexpressed, indicating the interest of overexpressing the transcriptional regulator to detect effectors secreted only in small quantities. Five of the 18 candidates were found not to be regulated by TtsI in the transcriptome analysis. Oppositely, 12 effectors whose gene expression is regulated by TtsI, including all the seven newly discovered TtsI-regulated candidates, were not found in the culture supernatant. Also 12 in silico predicted effectors were not detected by proteomics (Figure 2).

Among the 18 proteins detected only in WT and its TtsI-overexpressing derivative, 15 had been previously identified as putative effectors by in silico analyzes and 3 new candidates were identified: *BRAD3257_v2_7792, BRAD3257_v2_8013* and *BRAD3257_v2_8216* (Figure 2; Table S4, see Supplementary Materials). The transcriptome analysis indicated that these three genes have a TtsI-independent expression (Table S3, see Supplementary Materials), in agreement with the absence of *tts-boxes* in their promoter region. 

#### 3.2.4. Validation of T3SS-Effector Candidates Identified by Omics Using Western-Blotting

By combining the results of the transcriptomic and proteomic approaches as well as the data previously obtained by in silico analyzes, 37 putative effectors (regulated by TtsI, possessing a *tts*-box, secreted by the T3SS or similar to known effectors) were identified by one or more of these methods (Figure 2). The symbiotic role of effectors previously identified by bioinformatics has already been characterized [9]. In order to advance in the understanding of the mechanisms of the NF- independent, T3SS-dependent pathway, we aimed at characterizing newly identified putative effectors.

The transcriptomic and proteomic approaches have revealed ten candidates that may be new T3Es of the ORS3257 strain, but they have been validated by only one of the two approaches (Figure 2). In order to determine whether these proteins are secreted in a T3SS-dependent manner, strains producing six of these putative effectors with a histidine tag (His_6_) were constructed within the WT strain as well as the T3SS mutant. Subsequently, the presence of these proteins in the supernatant and the cellular content of ORS3257 strains was determined by Western blotting using an anti-histidine antibody.

Two proteins encoded by the genes *BRAD3257_v2_7760* and *BRAD3257_v2_8013* were not identified by Western blot both in the supernatants and in the pellets of the strains producing them, coupled with a His_6_-tag (Figure S2a, see Supplementary Materials). Three other candidate proteins encoded by the genes *BRAD3257_v2_7097*, *Brad3257_v2_7177* and *BRAD3257_v2_8216* were detected in the cell pellets but not in the supernatants of both the WT and T3SS mutant strains producing His_6_-tagged proteins (Figure S2a, see Supplementary Materials). This experience does not allow validating these five candidates as T3Es. In contrast, the protein encoded by the gene *BRAD3257_v2_7792* was identified in the cell pellets of the WT and T3SS mutant strains, but also specifically in the supernatant of the WT strain (Figure 3d). A control Western blot detecting the cytosolic GFP protein demonstrated the absence of cytosolic proteins in the culture supernatant due to cell lysis (Figure S2b, see Supplementary Materials). This result confirms the T3SS-dependent secretion of this protein and thus validates that *BRAD3257_v2_7792* codes for the synthesis of a T3E.

#### 3.2.5. Secretion of T3SS-Needle Components

Besides the effectors, the needle components of the T3SS machinery are also commonly found among the secreted proteins [30,31,32]. Two of the predicted T3SS-needle components, common to rhizobia, were found in the ORS3257 culture supernatant. The first one, NopA, which is the major constituent of the needle (pilin), is secreted in a T3SS-dependent manner (Table S4, see Supplementary Materials). The second identified component is NopX, which is the translocon, a translocation pore positioned at the tip of the T3SS needle allowing the passage of T3Es into the host cell (Table S4, see Supplementary Materials) [33,34]. It is interesting to note that NopX is apparently secreted in a T3SS-independent manner since the protein is also present in the supernatant of the T3SS mutant (Table S4, see Supplementary Materials). 

Another unexpected finding of the proteome study was the absence of detected secretion of NopB, which is one of the components of the T3SS-needle found in other phylogenetically related *Bradyrhizobium* species and *E. fredii* NGR234 (Table S4, see Supplementary Materials) [32,34,35]. Western blot analysis was used to detect NopB accumulation in the cells or supernatant of the wild strain, in order to confirm or not this surprising absence of the protein in the culture supernatant (Figure 3e). Using an anti-NopB antibody, it was possible to detect NopB in cells of the WT strain. However, the protein was not detected in the supernatant. Similarly, in a strain expressing NopB-His_6_, the tagged protein was only detected in the cell fraction and was absent from the culture supernatant, thus confirming the results obtained by proteomic analysis (Figure 3f).

### 3.3. A Mutant in the BRAD3257_v2_7792 Effector Is Not Impaired in Symbiosis with Aeschynomene indica

**Figure 4:**
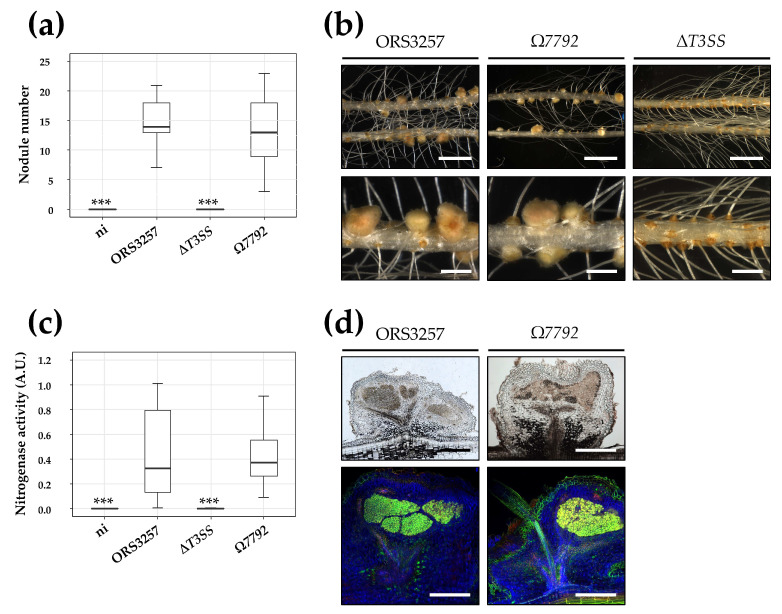
Symbiotic properties of the ORS3257Ω7792 mutant on *Aeschynomene indica*. (**a**) The number of nodules on *A. indica* plants at 21 days after inoculation with the WT strain ORS3257, the T3SS mutant (∆T3SS), and the *Brad3257_v2_7792* effector mutant (Ω7792). (**b**) View of the root and the nodules induced by strain ORS3257 and the ∆T3SS and Ω7792 mutants. Scale bars, 1.5 cm (upper panels) and 2 mm (lower panels). (**c**) Nitrogen fixation activity of *A. indica* plants at 21 days after inoculation with the WT strain ORS3257 and the ∆T3SS and Ω7792 mutants. A.U., arbitrary units. Box plots in (**a**,**c**) show results of one representative experiment out of at least two independent experiments per strain (12 plants each). The central rectangle spans the first quartile to the third quartile, the bold segment inside the rectangle shows the median, and the whiskers above and below the box show the locations of the maximum and minimum value, respectively. *** *p* < 0.001, significant differences between WT ORS3257 and the non-inoculated plants (ni) or the ∆T3SS mutant using a non-parametric Kruskal–Wallis test. (**d**) Cytological analysis of the nodules induced by strain ORS3257 and the Ω7792 mutant observed by light microscopy (upper panels) or confocal microscopy after staining with Syto9 (green; live bacteria), calcofluor (blue; plant cell wall), and propidium iodide (red; plant nuclei) (lower panels). Scale bars, 500 µm.

The validation by two different approaches that the BRAD3257_v2_7792 protein is secreted in a T3SS-dependent manner coupled with the fact that this protein contains a SUMO-protease domain similar to other type III effectors (Figure S3, see Supplementary Materials) suggests a role for this effector during symbiosis [36]. In order to determine if this effector is important during the NF-independent symbiotic process, *A. indica* plants were inoculated with the mutant ORS3257Ω*7792*, unable to produce this protein. Observation of nodules elicited by the mutant at 21dpi on the roots of *A. indica* did not show differences with those produced by the WT strain, either in the number of nodules or their appearance (Figure 4a,b). In addition, these nodules are perfectly infected and have a similar nitrogenase activity to those produced by the WT strain (Figure 4c,d). These results indicate that the mutation of BRAD3257_v2_7792 does not affect the symbiotic capacities of the ORS3257 strain during symbiosis with *A. indica* in the conditions of this experiment, suggesting that the BRAD3257_v2_7792 effector is dispensable during the NF-independent process.

## 4. Discussion

Legumes, through their symbiotic interaction with rhizobia, are of major agronomic and ecological importance. Understanding the molecular mechanisms allowing an optimal symbiosis between these two partners has been therefore of great interest for scientific research for many decades. Many rhizobial species use a T3SS to increase their host spectrum via the secretion of T3Es, sometimes called Nops for nodulation outer proteins that control immune responses in the host [37]. Characterizing the symbiotic role of T3Es could make it possible to optimize the symbiotic couples used in agronomy via a more detailed understanding of the molecular mechanisms supporting or preventing the symbiotic process. This requires, for each strain, identifying the cocktail of effectors secreted since certain effectors can act in synergy, have functional redundancies, or have opposing effects on the symbiotic process [23,38,39]. Several studies have already been carried out in order to produce catalogs of T3Es in *Bradyrhizobium* spp. or *E. fredii* strains using in silico, transcriptomic, or proteomic approaches [10,32,40,41]. A study carried out previously revealed that the *B. vignae* ORS3257 strain, in addition to having T3Es conventionally present in other rhizobia, also has a new class of effectors involved in the direct triggering of the symbiotic process [9]. ErnA is most probably the first discovered member of this type of effector, which we propose to call ET-Nods for effectors triggering nodulation. In order to characterize the effector catalog of this strain as precisely as possible and optimize the chances of identifying new T3Es or ET-Nods, we have combined in this study transcriptomic and proteomic approaches with the previously reported in silico analysis [9].

The combined approaches resulted in the identification of 36 putative T3Es validated by at least one of the methods. It should be noted that other candidates identified by transcriptomics or proteomics were not retained in this list because being isolated in the genome outside of the symbiotic island, but they could be the object of a further study. Within this list, thirteen putative effectors (NopM1, NopP1, NopT, Brad3257_v2_7172, Brad3257_v2_7178, NopM3, Brad3257_v2_7238, ErnA, NopAB, NopBW, NopAJ2, NopC, and NopP2) were validated by all of the used methods. They have a *tts-box* motif upstream of the genes, are under the control of TtsI and are secreted in a T3SS-dependent manner. In addition, for five of them, NopM1, NopP1, NopT, NopAB, and ErnA, mutation alters the symbiotic properties of the strain during the interaction with *A. indica* [9]. These proteins, most of which have homologs in other rhizobium species, are therefore undoubted T3Es.

Other candidates are not validated by all the methods. Some candidates have only been validated by bioinformatics analysis via homology with known T3Es in other species and/or by the presence of a *tts*-box that was found to be non-functional in the transcriptome analysis. Among them are NopAO, NopM2, NopAD, NopAC, NopBI, and NopAM. Except for NopM2, for which homologs were confirmed to be secreted via the T3SS in several rhizobia (including this study for NopM1 and NopM3), to our knowledge, no homolog of the other candidate effectors was proven to be a bona fide T3E in other rhizobia. The NopAO, NopAD, NopAC, NopBI, and NopAM proteins, initially identified in *Bradyrhizobium* strains by search of the *tts-*box motif, were proposed to correspond to T3Es using a heterologous *Pseudomonas–Arabidopsi*s translocation system [42]. In this system, the N-terminus of the candidate T3E is fused to the AvrRpt2 effector of *p. aeruginosa* lacking its own N-terminal secretion signal. The hybrid protein triggers an HR in *Arabidopsis* leaves if it is translocated by *Pseudomonas* [42]. However, this approach might generate many false positives since most of the tested candidates were positive in the assay, including several components of the basal T3SS apparatus, which are not expected to be secreted. Our study indicates that NopAO, NopAD, NopAC, NopBI, and NopAM should not be considered as rhizobial T3Es. However, this conclusion does not mean that these genes do not play a possible symbiotic role considering that a *nopAO* mutant in the ORS3257 strain was found to be positively impacted during symbiosis with *A. indica* [9].

On the other hand, some candidate genes initially identified by bioinformatics, such as *nopAJ1* and *nopAR* whose expression was not confirmed to be under the control of TtsI, were found to be secreted in a T3SS-dependent manner by proteomic analysis. Our initial hypothesis that the expression of the genes involved in the synthesis of T3SS and T3Es is strictly under the control of TtsI, is therefore not accurate and we have to propose that another system for regulating the expression of effectors exists in the ORS3257 strain, as has been reported in other strains [24,43]. 

Certain candidates, as for example NopAL, identified by bioinformatics and expressed under the control of TtsI, were not identified by proteomics. Too low abundance or instability of these potential effectors in the supernatant or during the precipitation of proteins could explain the absence of detection of these proteins. 

NopB is another undetected protein that was expected to be found in the supernatant of the T3SS-positive strains. Using three different strategies, by proteomics and by Western blot analysis with a specific anti-NopB antibody or with a His_6_-tag antibody, we could not detect the accumulation of this protein in the culture supernatant despite its detection in the cell fraction. NopB is considered as one of the major components of the T3SS needle, interacting with the NopA pilin, and classically found in the supernatant of rhizobia [32,35]. Possibly, NopB in ORS3257 is unstable in the culture supernatant, which would be in agreement with reported difficulties to detect NopB in a culture supernatant of *B. diazoefficiens* USDA110 [32,44]. Alternatively, the needle in strain ORS3257 has a different composition as compared to *E. fredii* and *B. diazoefficiens* USDA110 and, therefore, NopB has an altered and unknown function in the T3SS machinery. Besides NopB, also another anticipated needle component, NopX, behaved unexpectedly. NopX was detected in the culture supernatant, but its secretion was independent of a functional T3SS. This is unlike NopX secretion in the *E. fredii* strain NGR234, which is T3SS-dependent [29].

It is important to note that many proteins from the cytosol or membranes are present in the supernatant of the tested conditions. These proteins can hide, by their abundance, certain candidates. Our hypothesis is that these proteins can originate in part from OMVs produced by the strain since the majority of these proteins are found in the OMVs of other bacterial species [45,46]. However, it cannot be excluded that some proteins derive from cell lysis or cell leakage during the culture and the centrifugation steps. Another bias in the proteomic analysis is the modulation of proteins produced by the different mutants. In fact, a great variability is observed between the conditions in the protein content present in the supernatant, independently of the secretion by the T3SS. The regulator TtsI has been demonstrated in the NGR234 strain as regulating the synthesis of certain polysaccharides [47]. It is therefore possible that its overexpression in ORS3257 also modulates the metabolism of the bacteria and, therefore, the synthesis or export of proteins. The suppression or inactivation of the T3SS can also modulate the protein synthesis of bacteria, because the production of T3SS by bacteria has a high energy cost [48], thus explaining the variation in protein content between the tested strains. 

Finally, some candidates were only identified by transcriptomics or proteomics. The production of strains producing some of these candidates with a polyhistidine tag made it possible to test by a second biochemical method the validity of these candidates. Of the six tested, only one was validated by this method. These results demonstrate that a single approach, whether biochemical or by bioinformatics, does not allow us to identify with certainty a T3E. The coupling of the different approaches made in this study thus maximizes the chances of obtaining a robust effector catalog by eliminating false positives or negatives produced by one of the used methods. Nevertheless, to conclusively qualify identified candidates as genuine T3Es, it would be necessary to use additional bio-assays to show their translocation into host cells.

BRAD3257_v2_7792, although not validated by transcriptomics, was identified by proteomics and Western blotting. In addition to its T3SS-dependent secretion, the fact that this protein contains a ubiquitin-like protease (ULP)-like domain, conserved in eukaryotic SUMO proteases and many bacterial effectors, makes it a very robust candidate for being a true T3E [49,50]. Protein desumoylation catalyzed by this type of enzyme controls cellular mechanisms ranging from transcription and cell division to ribosome biogenesis [27]. Presently, only two SUMO-protease rhizobial effectors identified in *Bradyrhizobium* strains were shown to impact positively or negatively on the symbiosis depending on the host plant [8,51,52]. One of them, the Bel2–5 effector secreted by *B. elkanii* USDA61, was also shown to be an ET-Nod since it acts as the main inducer of the T3SS-dependent symbiosis on Glycine max cv. Enrei [8,53]. Nevertheless, the *BRAD3257_v2_7792* mutant strain did not show a detectable phenotype during symbiosis with *A. indica*. However, since effectors have host-specific activities, it is likely that this effector is involved in the interaction with another host plant. Considering that *B. vignae* is also able to nodulate with a NF-dependent process other legume species such as *Vigna unguiculata* or *Arachis hypogaea* [54,55], we can speculate that this effector plays a role during the symbiotic interaction with other host legume species.

In conclusion, this study produced a catalog of candidate T3Es of the *B. vignae* ORS3257 strain and confirmed previously identified effectors whose role during symbiosis with *A. indica* and three *Vigna* species has been determined already [9,41]. The here refined catalog of effectors will allow the comprehensive analysis of the impact of the T3SS and its effectors in symbiosis with *A. indica*, or other species nodulated in a T3SS-dependent manner, or with other legume hosts such as *Vigna* spp*. or Arachis hypogea*, in order to deepen our understanding of this process. Many other strains of *Bradyrhizobium* use a T3SS-dependent symbiosis [7]. The identification and characterization of the cocktails of effectors produced by these strains will allow us to advance in the characterization of this new symbiotic pathway and perhaps to discover new ones via the identification of novel ET-Nod effectors such as ErnA and Bel2–5. Thirteen years ago, the Nod factors were considered to be essential to all rhizobium-legume symbioses, today two NF-independent symbiosis pathways have been identified. How many tomorrow?

## Data Availability

The sequencing data presented in this study are openly available in the Sequence Read Archive (SRA) with reference number n° SRR14479858 to SRR14479861.

## Supplementary Materials

The following are available online at https://www.mdpi.com/article/10.3390/biom11111592/s1, Figure S1: RNA-seq read quality; Figure S2: Western blot analysis of putative effectors in cell-associated and supernatant proteins of ORS3257 WT and ∆T3SS strains; Figure S3: Similarity between the BRAD3257_v2_7792 effector and two other rhizobial effectors containing a SUMO-protease domain; Table S1: List of strains and primers used in this study; Table S2: ORS3257 genes overexpressed in the WT condition compared to the Ω*ttsI* condition under genistein induction; Table S3: DeSeq2 analysis of the transcriptome data set; Table S4: Proteins detected in culture supernatants of ORS3257, the T3SS mutant and derivatives overexpressing TtsI.
