# Supplementary material for: The Type III Effectome of the Symbiotic Bradyrhizobium vignae Strain ORS3257
Source: Biomolecules. 2021 Oct 28;11(11):1592. doi: 10.3390/biom11111592 (PMC8615406; doi:10.3390/biom11111592)
Supplement: Supplementary file 1 [file biomolecules-11-01592-s001.zip › Figure S1.pdf]

(a)
(b)
(c)
(d)
(e)
(f)
(g)
(h)

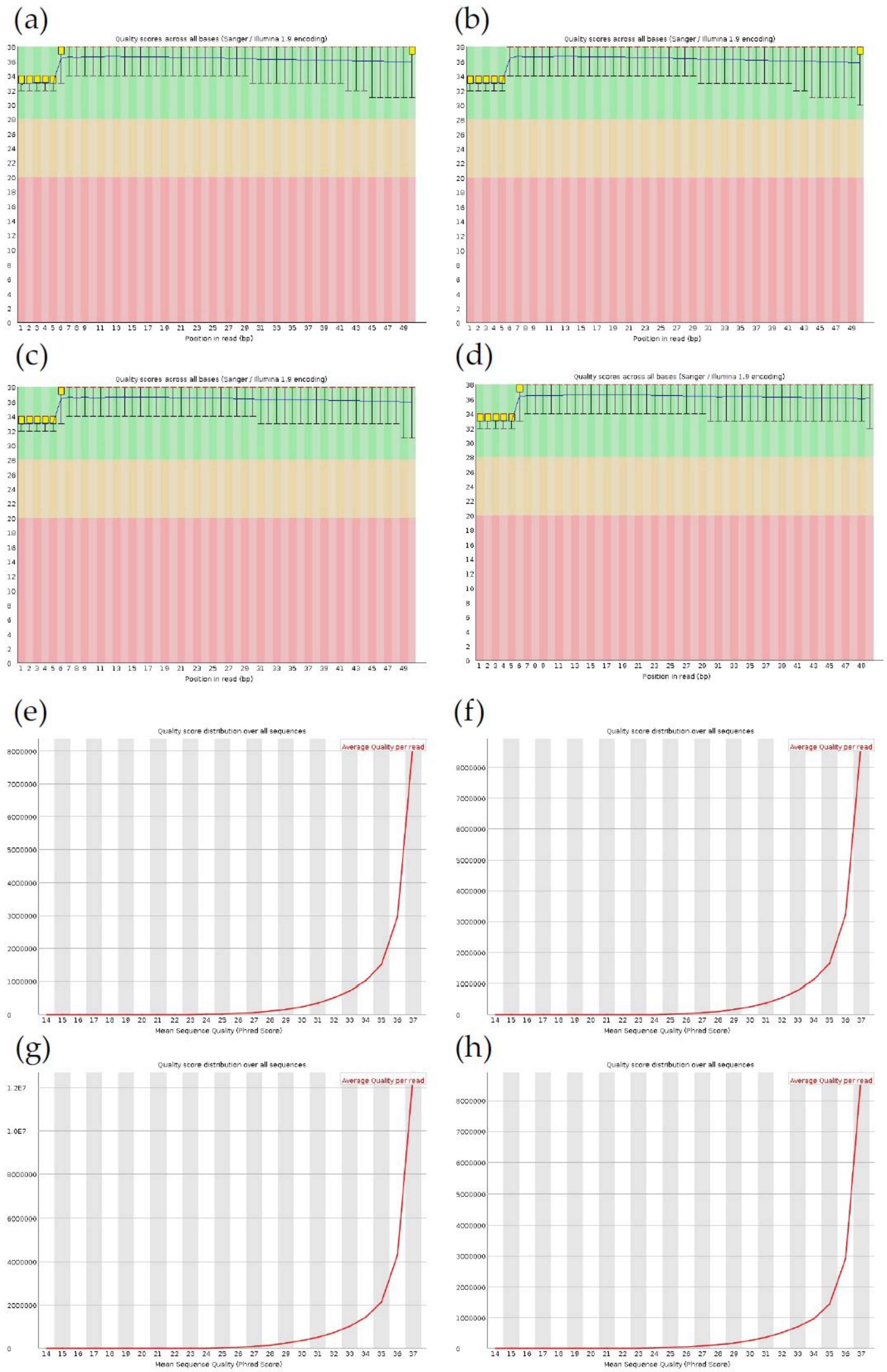

Figure S1. RNA-seq read quality. **(a-d)** Phred quality per base. **(e-h)** Mean quality distribution. Inserts a /e, b/f, c/g and d/h correspond respectively to SRA accession SRR14479858 to SRR14479861.
