# Supplementary material for: The Type III Effectome of the Symbiotic Bradyrhizobium vignae Strain ORS3257
Source: Biomolecules. 2021 Oct 28;11(11):1592. doi: 10.3390/biom11111592 (PMC8615406; doi:10.3390/biom11111592)
Supplement: Supplementary file 1 [file biomolecules-11-01592-s001.zip › Figure S2 revised2.pdf]

(a)

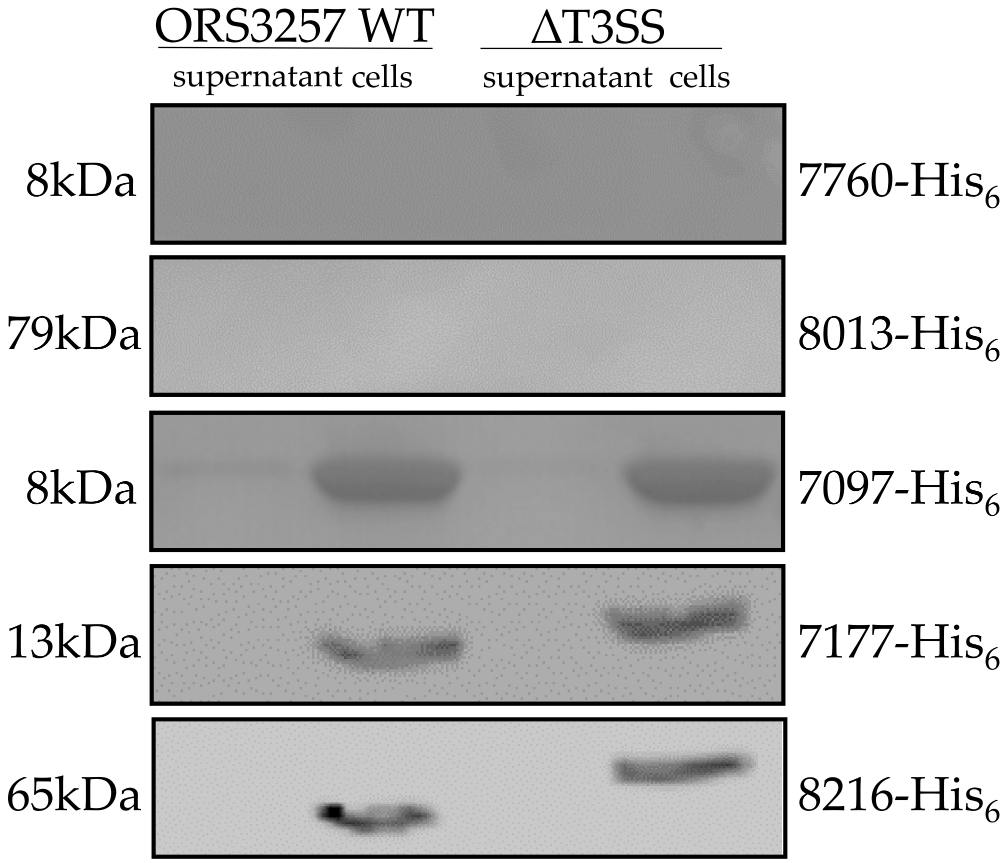

(b)

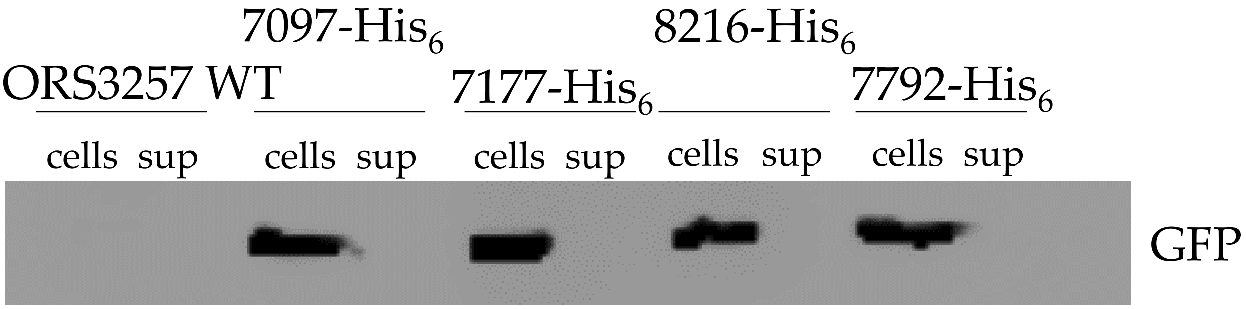

Figure S2. Western blot analysis of putative effectors in cell-associated and supernatant proteins of ORS3257 WT and ΔT3SS strains. (**a**) Western blots were performed with an anti-His6 antibody. The gene accession number corresponding to the tagged protein for each strain is indicated on the right and the size of the band on the left. Note the size differences for the 7177-His$_6$ and 8216-His$_6$ proteins in the cell extracts of the WT strain and ΔT3SS mutant, which are likely due to distortions in the migration profile during the gel electrophoresis. (**b**) Western blot with anti-GFP antibody of cells and supernatant (sup) proteins of ORS3257 WT and strains producing His6 tagged proteins. The gene number corresponding to the tagged protein for each strain is indicated.
