# Supplementary material for: The Type III Effectome of the Symbiotic Bradyrhizobium vignae Strain ORS3257
Source: Biomolecules. 2021 Oct 28;11(11):1592. doi: 10.3390/biom11111592 (PMC8615406; doi:10.3390/biom11111592)
Supplement: Supplementary file 1 [file biomolecules-11-01592-s001.zip › Figure S3 modified_pm.pdf]

(a)

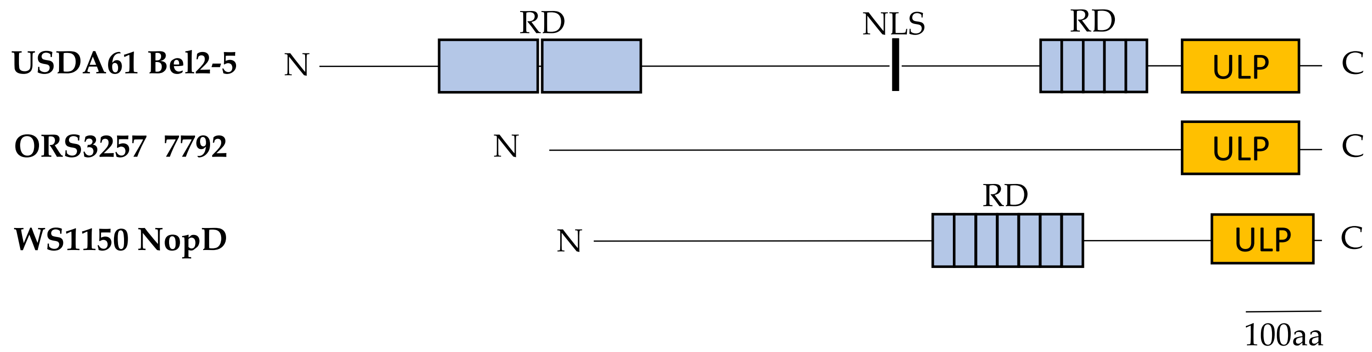

(b)

```
7792   WLGDEHIQRDYELLAQELWASNPNLAARTRFADPLTAF-RVGQGTNAAALSAFHQIVDDPNGNDTADFLFLPVN
Bel2-5 WLGDEHIQRDYELLAQELWASNPNLAARTRFADPLTALNYLRSNDDGVVLTEFQRIVYDDNGNDTADFLFLPVI
NopD   WLGDEHIQRDYELLAQELWASNPNLAARTRFADPLTAQ-MLRSPSKEVAERALGWVR--PG---TADFLFLPVS

7792   DASTMDLDDRGSHWSLLLVDRRDRNNPVAYHYDSARGRNDRPAAMLAARVGAGDNLQNAPIRQQRNGYDCGVFV
Bel2-5 NGNPEDPNSRGNHWSLLFVDRSDRWRPVAYHYDSYGGLNNRDAAHLARRLNLP--LELADMAQQQNTYDCGVFV
NopD   DASDTDRHQRGSHWSLLLVDRRDRGRRVAYHYDSTQGYNDGLAAELAGRLDA--NLQQAPIRQQQNSYDCGVFV

7792   LDGTR---------------------------
Bel2-5 VDGTRELVRQLAQGWEPDQLNLSNVVANRQ--
NopD   LDGTRELVRRLAA--RRPDLNLNNLVISRQEL
```

Figure S3. Similarity between the BRAD3257_v2_7792 effector and two other rhizobial effectors containing a SUMO-protease domain. (**a**) Schematic representation of the effectors BRAD3257_v2_7792 (7792) from *B. vignae* ORS3257, Bel2-5 from *B. elkanii* USDA61, and NopD from *Bradyrhizobium* sp. XS1150. RD is for repeat domain; NLS is for Nuclear Localization Sequence; ULP is for ubiquitin-like protease (SUMO protease domain). (**b**) Sequence alignment of the ULP domains.
